# A chelicerate-specific burst of nonclassical Dscam diversity

**DOI:** 10.1186/s12864-017-4420-0

**Published:** 2018-01-19

**Authors:** Guozheng Cao, Yang Shi, Jian Zhang, Hongru Ma, Shouqing Hou, Haiyang Dong, Weiling Hong, Shuo Chen, Hao Li, Yandan Wu, Pengjuan Guo, Xu Shao, Bingbing Xu, Feng Shi, Yijun Meng, Yongfeng Jin

**Affiliations:** 1 Zhejiang University, Institute of Biochemistry, College of Life Sciences, Hangzhou, Zhejiang ZJ310058 People’s Republic of China; 2 College of Life and Environmental Sciences, Hangzhou Normal University, Hangzhou, Zhejiang ZJ310018 People’s Republic of China

**Keywords:** Dscam, Gene duplication, Exon duplication, Alternative splicing, Alternative promoter, Chelicerate

## Abstract

**Background:**

The immunoglobulin (Ig) superfamily receptor Down syndrome cell adhesion molecule (*Dscam*) gene can generate tens of thousands of isoforms via alternative splicing, which is essential for both nervous and immune systems in insects. However, further information is required to develop a comprehensive view of *Dscam* diversification across the broad spectrum of Chelicerata clades, a basal branch of arthropods and the second largest group of terrestrial animals.

**Results:**

In this study, a genome-wide comprehensive analysis of *Dscam* genes across Chelicerata species revealed a burst of nonclassical *Dscam*s, categorised into four types—*mDscam*, *sDscamα*, *sDscamβ*, and *sDscamγ*—based on their size and structure. Although the *mDscam* gene class includes the highest number of *Dscam* genes, the *sDscam* genes utilise alternative promoters to expand protein diversity. Furthermore, we indicated that the 5′ cassette duplicate is inversely correlated with the *sDscam* gene duplicate. We showed differential and *sDscam*- biased expression of nonclassical Dscam isoforms. Thus, the Dscam isoform repertoire across Chelicerata is entirely dominated by the number and expression levels of nonclassical *Dscam*s. Taken together, these data show that Chelicerata evolved a large conserved and lineage-specific repertoire of nonclassical Dscams.

**Conclusions:**

This study showed that arthropods have a large diversified Chelicerata-specific repertoire of nonclassical Dscam isoforms, which are structurally and mechanistically distinct from those of insects. These findings provide a global framework for the evolution of Dscam diversity in arthropods and offer mechanistic insights into the diversification of the clade-specific Ig superfamily repertoire.

**Electronic supplementary material:**

The online version of this article (10.1186/s12864-017-4420-0) contains supplementary material, which is available to authorized users.

## Background

Alternative splicing plays an important role in the generation of proteomic diversity and genomic evolution in metazoans [1, 2]. For example, the Down syndrome cell adhesion molecule (*Dscam*) gene in *Drosophila melanogaster* has the potential to generate 38,016 distinct mRNA and protein isoforms via mutually exclusive alternative splicing [3]. In this *Dscam* gene structure, 95 alternatively spliced exons are organised into exon 4, 6, 9, and 17 clusters that contain 12, 48, 33, and 2 copies, respectively. Forward genetic screening and biochemical approaches have shown that this extensive diversity of Dscam encoded by a single locus is required for both nervous and immune systems. Dscam isoform diversity plays an important role in neuronal wiring and self-recognition, and the extensive diversity of Dscam isoforms has been shown to confer specificity for antigen recognition [4–13].

Vertebrate Dscams, which lack the striking diversity of their insect counterparts, are involved mainly in the developmental processes of the nervous system [14]. Interestingly, cadherin superfamily members, protocadherins (Pcdhs), might serve an analogous function in vertebrates [15–17]. Human *Pcdh* genes are arranged tandemly in three groups called *Pcdh*α, *Pcdhβ*, and *Pcdhγ*, with 14, 22, and 22 repeats in their respective 5′ variable regions [18]. Unlike *Drosophila Dscam1*, these *Pcdh*s utilise alternative promoters to generate isoform diversity [19, 20]. For both Dscams and Pcdhs, isoform expression appears largely stochastic and combinatorial rather than determinative, endowing a unique cell-surface identity for each neuron [15, 16, 21, 22]. Both molecules exhibit isoform-specific homophilic binding, and they may have similar roles in the nervous system. Interestingly, *Drosophila* lacks the counterparts of vertebrate *Pcdh*s. Thus, these two phyla appear to have independently evolved similar molecular strategies for comparable roles by recruiting various molecules from different protein families [15].

As Pcdh isoform diversity is restricted to vertebrates, Dscam isoform diversity has been considered unique to arthropods [23, 24]. However, compared with the well-known phylogenetic distribution of vertebrate *Pcdh*s, *Dscam* diversification has not been studied in detail in arthropods, particularly in Chelicerata members, which represent a basal branch of arthropods and form the second largest group of terrestrial animals. It is generally recognised that Dscam protein structure is conserved across bilaterians, containing 10 immunoglobulin (Ig) domains and six fibronectin III (FNIII) repeats, with the tenth Ig domain located between FNIII 4 and FNIII 5 [14, 15, 17]. Recently, structural variants of Dscam have been found in *Ixodes scapularis* [24]. We identified shortened *Dscam* genes with tandemly arrayed 5′ cassettes in several Chelicerata species, similar to vertebrate clustered *Pcdh*s [25]. These results suggest that the structural and expansion pathways of *Dscam* might be diversified in Chelicerata species. However, no comprehensive view exists of *Dscam* diversification across the broad spectrum of Chelicerata. The rapidly increasing availability of genomic information regarding Chelicerata, particularly key clades such as the Xiphosuran horseshoe crab, will increase our understanding of Dscam diversification.

In this study, we performed a genome-wide comprehensive analysis of *Dscam* genes in chelicerates, which diverged from other Arthropod lineages ~500 million years ago. The identified *Dscam* genes could be grouped into one classical (*LDscam*) and four nonclassical (*mDscam*, *sDscamα*, *sDscamβ*, and *sDscamγ*) types based on their size and structure. Although the *mDscam* gene class includes the highest number of *Dscam* genes, the *sDscam* genes utilise alternative promoters to expand protein diversity. Collectively, these results demonstrate that Chelicerata specifically evolved different organisation and mechanisms that generated a diverse lineage-specific repertoire of Dscam isoforms. These findings highlight the rich Chelicerata-specific diversification of *Dscam* genes, and provide a global framework for the evolution of Dscam diversity in arthropods and bilaterians.

## Results

### Genome-wide identification of *Dscam* genes across Chelicerata species

**Fig. 1:**
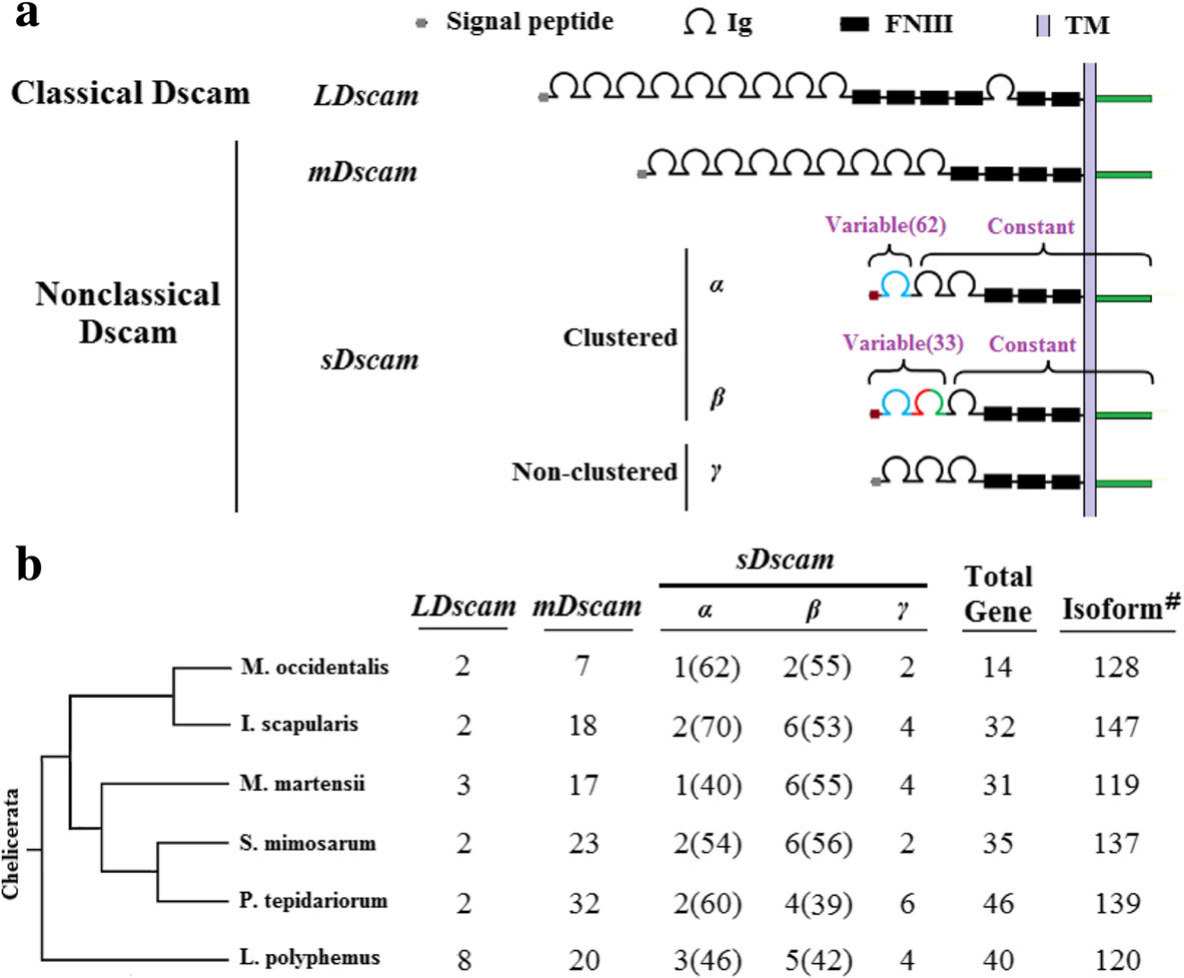
Genome-wide identification and classification of *Dscam*s in Chelicerata. **a** Schematic representation of Dscam structures in Chelicerata. Ig, immunoglobulin domains; FNIII, fibronectin III domains. The N-terminal small boxes represent the leader peptides. The lavender blue and green boxes represent the transmembrane (TM) and cytoplasmic domains. Five *Dscam* types (*LDscam*, *mDscam*, *sDscamα*, *sDscamβ*, and *sDscamγ*) are classified based on their size and structure. *LDscam* shares structures identical to classical *Dscam*. *mDscam* lacks Ig10 and FNIII 3–4 domains of classical *Dscam. sDscamα* contains variable N-terminal IgI (blue), which corresponds to the variable Ig7 domain of *Drosophila Dscam1*. *sDscamβ* contains variable N-terminal Ig1 + 2 domains (coloured), which correspond to the variable Ig7 + 8 domains of *Drosophila Dscam1*. Numbers in parentheses refer to the numbers of 5′ variable cassettes in *Metaseiulus occidentalis sDscamα* and *sDscamβ2*. *sDscamγ* shares domains similar to *sDscamα* and *sDscamβ*, albeit with no tandemly arrayed cassettes in the 5′ regions. Numbers in parentheses refer to the numbers of tandem cassettes. **b** Phylogenetic distribution of *Dscam*s and isoform members in chelicerates. Dscams are shown associated with a cladogram of phylogenetic relationships in this study [52]. # Indicates the putative numbers of Dscam isoforms in various arthropod species caused by either gene and/or exon duplication, estimated by the number of Ig7 or orthologues

To generate a global blueprint for *Dscam* diversity in Chelicerata, we performed a genome-wide analysis of *Dscam* homologues in representative species from each of the major clades. We examined one species of the order Xiphosura (*Limulus polyphemus*) and five species representing five major clades of the class Arachnida, including two Araneae (*Stegodyphus mimosarum* and *Parasteatoda tepidariorum*), one Scorpiones (*Mesobuthus martensii*), one Mesostigmatan (*Metaseiulus occidentalis*), and one Ixodidan (*Ixodes scapularis*) (Additional file 1: Table S1)*.* These organisms constitute some of the major taxonomic groups of the Chelicerata subphylum that last shared a common ancestor ~500 million years ago [26], with long-term resolution (comparing the gene organisation among the five Arachnida clades and Merostomata). Using cross-species comparisons with RNA-sequencing (RNA-seq) analyses, we identified 198 *Dscam* genes in six representatives of the Chelicerata species, 161 of which were novel or corrected (Fig. 1b; Additional file 2: Table S2). Our results indicated that all extant chelicerates display marked expansion of the *Dscam* gene family.

**Fig. 2:**
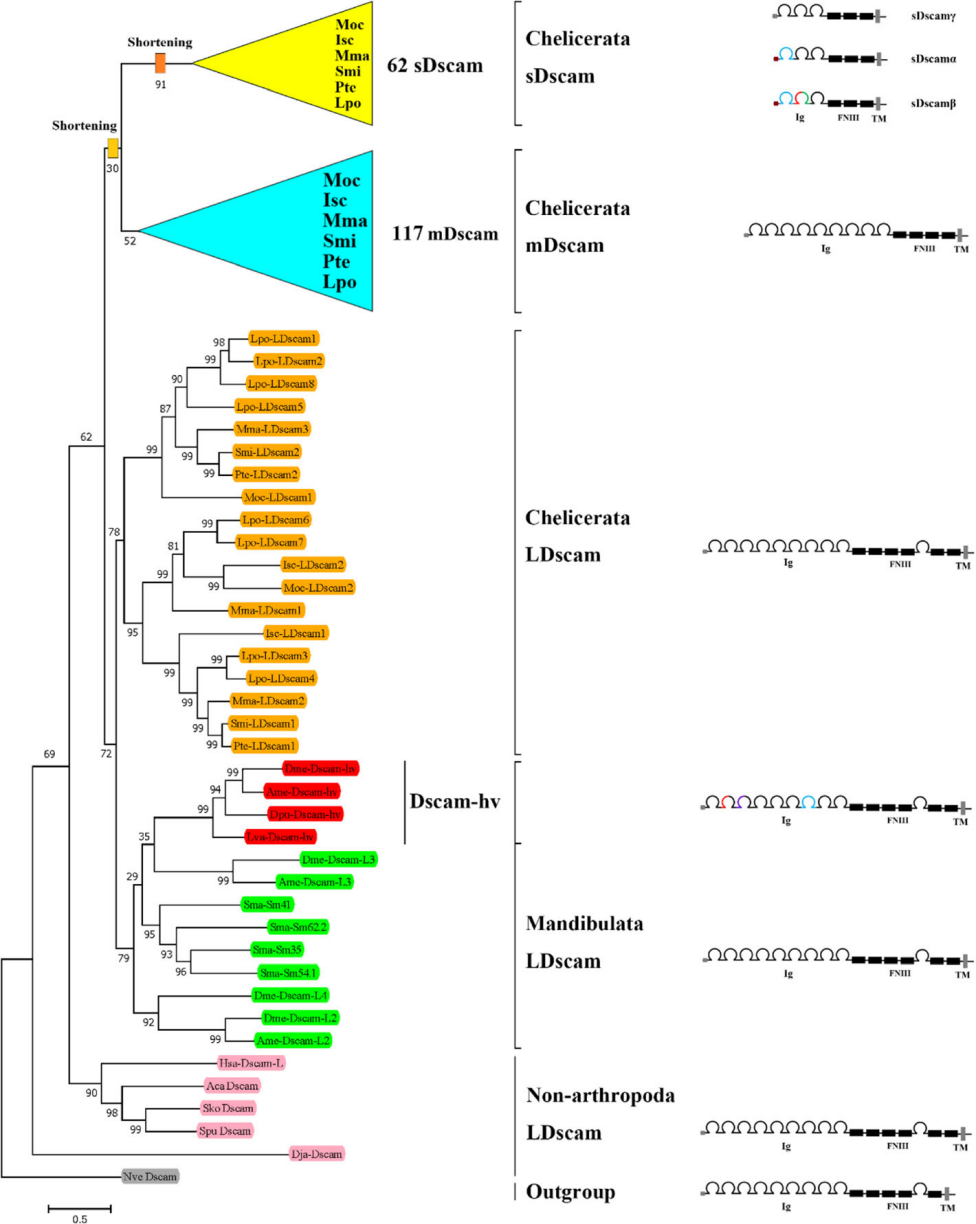
The evolutionary relationships and protein structures of arthropoda Dscams. The tree is based on amino acid sequence alignment of Dscams encompassing the seventh Ig domains to end (sDscam regions encompassing the first Ig domains to end), and is rooted using the sequence of the *Nematostella vectensis* Dscam (GenBank:ABAV01020293.1) [51]. The support values at the nodes are bootstra*p* values relative to 1000 replicates. We considered five *Dscam* types (*LDscam*, *mDscam*, *sDscamα*, *sDscamβ*, and *sDscamγ*) in Chelicerates, which are detailed in Additional file 2: Table S2. *L. vannamei* (Lva) *Dscam1* (GQ154653), *D. melanogaster* (Dme) *Dscam1*–*4* (CG17800; CG42256; CG31190; CG42330), *A. mellifera* (Ame) *Dscam* (AAT96374; BAF03050.1; XM_396307), *D. pulex Dscam1* (EU307884), *A. californica* (Aca) *Dscam* (ABS30432.1), *S. kowalevskii* (Sko) *Dscam* (XP_006825869.1), *S. purpuratus* (Spu) *Dscam* (XP_011665747.1), *D. japonica* (Dja) *Dscam* (BAE94189.1), and *H. sapiens* (Hsa) *Dscam-L* (AAL57166.1) are included. For other canonical *Dscam* sequences from *S. maritima*, refer to recent references [24]. We collapsed the monophyletic clades of sDscams and mDscams for visualization convenience. Their detailed phylogenetic relationships are shown in Additional file 3: Figures S1 and S2, respectively. The sequential shortening of the Ig and FNIII domains of canonical *Dscam* is indicated by orange squares

Phylogenetic analysis indicated that all of the Dscam proteins could be clustered into three groups: canonical Dscams and two groups of shortened nonclassical proteins (Fig. 2). The three groups differ in size, and are hereafter referred to as LDscam, mDscam, and sDscam, respectively (Fig. 1a). The sDscams lack the canonical Ig1–6,10 and FNIII 3–4,6 domains present in classical Dscam. The genes encoding these sDscams can be subdivided into three types—*sDscamα*, *sDscamβ*, and *sDscamγ*—based on differing structures in the 5′ regions (Fig. 1a)*. sDscamα* and *sDscamβ* are characterised by clustered cassette repeats in the 5′ regions, encoding one and two Ig domains, respectively. *sDscamγ* shares similar domains with *sDscamα* and *sDscamβ*, albeit without tandemly arrayed cassettes in the 5′ regions. Another type of shortened Dscam lacks the FNIII and Ig domains present in the C-terminal region of classical Dscam. As the sizes of these Dscams fall between those of canonical Dscam (LDscam) and sDscam, we designated these intermediate shortened *Dscam* genes as *mDscam*s. As shown in Fig. 1b, nonclassical Dscams dominate the isoform repertoire in all Chelicerata species investigated.

### Nonclassical *Dscam* genes are Chelicerata-conserved and restricted

This study revealed that nonclassical *Dscam*s are conserved across Chelicerata species and that all types of *sDscam*s (*sDscamα*, *sDscamβ*, and *sDscamγ*) are largely present in representative species from the Arachnida and Merostomata classes investigated in this study (Fig. 1b). Our results suggest that these types of nonclassical *Dscam*s are ancient, existing before the split of Arachnida and Merostomata. The *Dscam* gene family members diverged markedly across various species. The higher gene number in scorpions, spiders, and horseshoe crabs is consistent with an additional event of whole or large-scale genomic duplications [27]. Although *mDscam* genes are present in greater numbers than *LDscam*s and *sDscam*s, the *sDscam* genes can generate up to a hundred isoforms through a combination of alternating promoters and alternative splicing. Thus, based on the isoform number, *sDscam*s are represented more than *LDscam*s and *mDscam*s in each Chelicerata. However, no such nonclassical *Dscams* have been identified among the *Dscam* genes from the Mandibulata species of insect, Crustacea, or Myriapoda classes, or in any non-arthropod species. This observation suggests that they arose after the radiation of Mandibulata and Chelicerata during Arthropoda evolution. Thus, we conclude that the nonclassical *Dscam*s are largely conserved and restricted to Chelicerata.

### Global analysis of *Dscam* relationships over the Chelicerata phylogeny

To trace the evolutionary history of nonclassical *Dscam* genes, we first performed multiple sequence alignments of all *Dscam*s from six representative species. Comparative analysis of *Dscam* sequences from Chelicerata and outgroup species revealed three major clades, which represent three groups of *LDscam*, *mDscam*, and *sDscam* (Fig. 2). Based on the phylogenies of individual *Dscam* types (Additional file 3: Figures S1 and S2), although our analyses of the *Dscam* genes did not permit a full ancestral reconstruction, several conclusions could be reached. First, these data indicate that at least seven *mDscam*s and five *sDscams* were present in the Chelicerata ancestor before the split of Arachnida and Merostomata (Additional file 3: Figures S1 and S2). Second, three types of *LDscam*s, *mDscam*s, and *sDscam*s were inconsistently expanded. *mDscam* genes have undergone massive duplications during Chelicerata evolution, while *LDscam* genes have undergone few or limited duplications (Fig. 2; Additional file 3: Figures S1 and S2). For example, the divergence of the Araneae ancestor into the *P. tepidariorum* and *S. mimosarum* ancestors involved 10 *P. tepidariorum*-specific duplications of *mDscam* (Additional file 3: Figure S1). This gene expansion process is ongoing, as demonstrated by recent duplications in the Araneae species.

Phylogenetic analysis revealed that *sDscam*s could be clustered into four clades (clades A−D; Additional file 3: Figure S2). Clade D exclusively included *sDscamβ*s from all species investigated, suggesting this *sDscamβ* is ancient and arose in the Chelicerata ancestor. Clade B consisted of conserved *sDscamα*s and species-specific *sDscamγ*, suggesting that this *sDscamα* arose from the Chelicerata ancestor. Interestingly, clade C included species-specific *sDscamβ*s and *sDscamα*s, in addition to *sDscamγ*s. We speculate that an *sDscamγ* ancestor evolved differentially into *sDscamβ*s or *sDscamα*s during Chelicerata divergence. These results indicated that *sDscamβ*s and *sDscamα*s might have multiple independent origins.

### A lineage-specific burst of 5′ clustered cassettes in *sDscam* genes

**Fig. 3:**
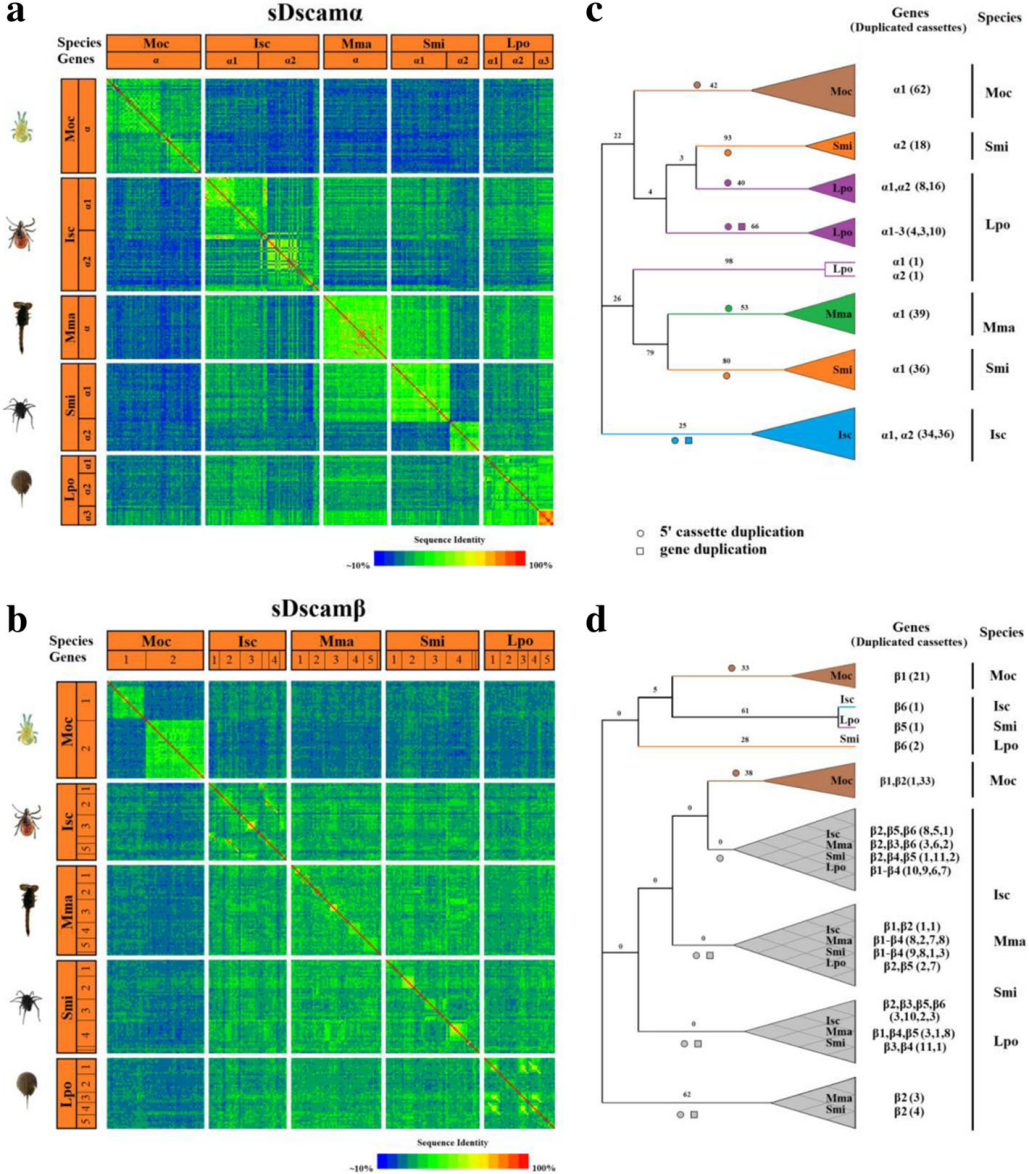
Similarities among 5′ variable cassettes of *sDscam* genes in representative species from each of the major Chelicerata clades. **a** Heatmap of pairwise sequence identities of the duplicated cassettes encoding the Ig1 domain of sDscamα isoforms from *Metaseiulus occidentalis* (Moc), *Ixodes scapularis* (Isc), *Mesobuthus martensii* (Mma), *Stegodyphus mimosarum* (Smi), and *Limulus polyphemus* (Lpo). To simplify visualisation, the cassette repeats are depicted in the heatmaps in the same linear order in which they reside in each gene. **b** Heatmaps of the percent identities of the duplicated cassettes encoding Ig1 + 2 domains of sDscamβ in Chelicerata species. The order of the cassettes in the heatmap corresponds to the linear order of each cassette in the genome. **c** Phylogenetic tree for the 268 duplicated cassettes encoding Ig1 domains of sDscamα. The support values at the nodes are bootstrap values relative to 1000 replicates. Numbers in parentheses refer to the number of 5′ variable cassettes of the corresponding gene. Clades were collapsed for visualisation convenience. **d** Phylogenetic tree for the 243 duplicated cassettes encoding Ig1 + 2 domains of sDscamβ. Collapsed clades plus crosses are depicted as a combined clade composed of these duplicated cassettes from different species

To produce an overview of the evolutionary relationships among the variable cassettes, we generated heatmaps for *sDscamα* and *sDscamβ* to show the relative similarities of each cassette repeat to other variable repeats both within and between species. For these analyses, we selected one representative species from each of the major orders investigated: *M. occidentalis*, *S. mimosarum*, *M*. *martensii*, *I*. *scapularis*, and *L. polyphemus*. Analysis of the heatmaps of the tandemly arrayed 5′ cassettes in the *sDscamα*s and *sDscamβ*s revealed little evidence of conserved orthologous pairs of repeats between species (Fig. 3a, b). Instead, when striking similarities were found between repeats in each species, they typically involved large blocks of highly similar cassettes within each gene of one species. For example, a massive block of 62, 36, and 18 cassettes expanded specifically in *sDscamα* of *M. occidentalis*, and in *sDscamα1* and *sDscamα2* of *S. mimosarum*, respectively (Fig. 3c). Similarly, blocks of 21 and 33 cassettes in the *M. occidentalis sDscamβ1* and *sDscamβ2*, respectively, were highly similar to one another (Fig. 3b, d). Phylogenetic analysis of duplicated cassettes from two closely related spiders (*S. mimosarum* and *P. tepidariorum*) indicated that 5′ cassette duplication largely occurs in a lineage-specific manner (Additional file 3: Figure S3). Furthermore, duplicated cassettes showed the tendency to be located adjacent to one another within a gene. Overall, these data indicate that the main expansions of ancestral cassettes of *sDscamα* and *sDscamβ*s occurred independently in each lineage.

The heatmap patterns differed considerably between *sDscamα*s and *sDscamβ*s among the various species. The *sDscamα* heatmap revealed large blocks of cassette duplications specific to each clade, consistent with phylogenetic analyses of duplicated cassettes across Chelicerata species (Fig. 3c). In contrast, with the exceptions of *M. occidentalis*, most *sDscamβ*s contain a number of common blocks that span multiple clades, as represented in green shading in Fig. 3b*.* Notably, duplications of 5′ cassettes in *sDscamα*s and *sDscamβ*s are almost exclusively specific to *M. occidentalis*, showing fewer similarities with other cassettes from other species, as represented by blue blocks in the regions of the heatmap that compare two species (Fig. 3a, b). Such high species specificity of the 5′ variable cassettes suggests that *sDscamα*s and *sDscamβ*s have undergone rapid expansions and divergence. Furthermore, comparison of 5′ cassette- and gene-based clustering clearly indicated that 5′ cassette duplication occurs on a much faster timescale than gene duplication (Additional file 3: Figure S4). This multi-layer expansion of sDscam diversity might help to increase the efficiency and flexibility of spatiotemporal regulation.

**Fig. 4:**
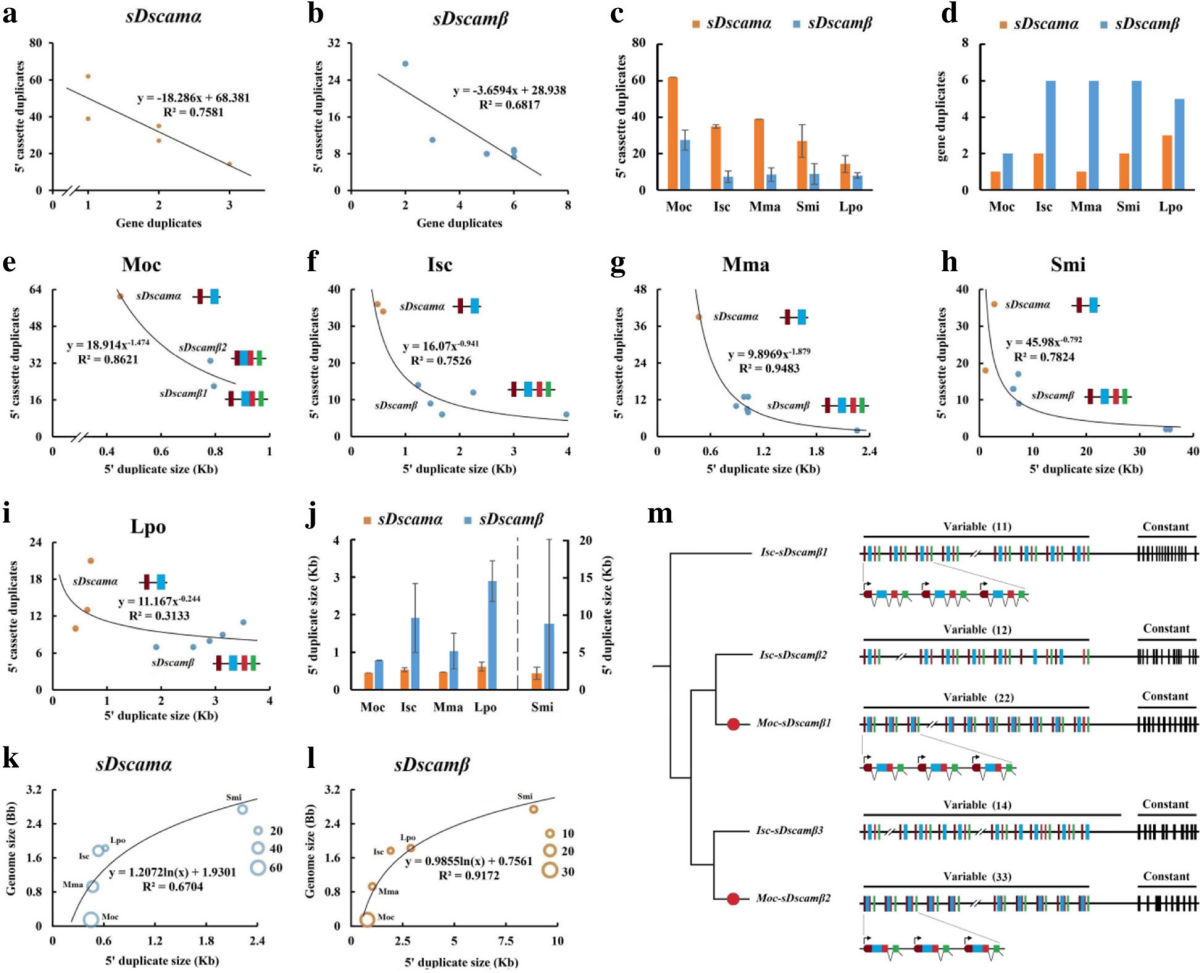
Inverse relationship between the number of 5′ cassette tandem duplicates and gene duplicates. **a, b** The number of 5′ cassette duplicates correlated inversely with the number of gene duplicates in *sDscamα* (a) and *sDscamβ* (b) in Chelicerata species. **c, d** Comparison of the number of 5′ cassette (c) and gene duplicates (d) between *sDscamα* and *sDscamβ*. Data are expressed as mean ± standard deviation (SD). **e−i** The number of 5′ cassette tandem duplicates correlated inversely with the duplicate size. This reverse relationship is conserved in *M. occidentalis* (e), *I*. *scapularis* (f), *M*. *martensii* (g), *S. mimosarum* (h), and *L. polyphemus* (i). **j** Comparison of the size of cassette repeats in *sDscamα* and *sDscamβ*. **k, l** The size of the cassette repeats correlated with the genome size of Chelicerata in *sDscamα* (k) and *sDscamβ* (l). The number of 5′ cassette tandem duplicates is indicated by the circle size. **m** Intron loss occurred independently in the *sDscamβ1* and *sDscamβ2* genes of *M. occidentalis*. The introns are represented with lines (not drawn to scale). Red circles indicate the intron loss, and the arrows indicate the transcription start sites

### 5′ cassette duplicate inversely correlated with *sDscam* gene duplicates

As gene duplications and 5′ cassette tandem duplications increased isoform diversity through the emergence of additional genomic copies, we next investigated whether or how they are related to each other as evolutionary mechanisms. We found that the number of 5′ cassette tandem duplicates correlated inversely with that of gene duplicates in the *sDscamα* and *sDscamβ* subfamily (Fig. 4a, b). Single *sDscam*s (singletons) are likely to contain substantially more tandem cassettes in the 5′ variable region. For example, up to 62 and 40 copies reside in the 5′ variable region of *sDscamα* in *M. occidentalis* and *M*. *martensii*, respectively. Furthermore, the *sDscamα* subfamily (1–3 members) contains two- to four-fold more 5′ cassette duplicates per gene, which is similar to members in the larger *sDscamβ* subfamily (2–7 members) (Fig. 4c); we found the reverse trend in gene duplicates (Fig. 4d). Importantly, the total number of Dscam isoforms is roughly similar among various Chelicerata species (Fig. 1b). Therefore, this correlation may reflect compensatory evolution between alternative promoter and gene duplication, analogous to the inverse correlation of alternative splicing with gene duplication [28]. These results imply that the inverse correlation is not simply an inherent inclination, but is instead fulfilling the complementary demand for expanding Dscam isoforms via distinct evolutionary mechanisms.

To further examine the evolutionary forces underlying the inverse correlation, we carried out a detailed comparison of duplication scenarios in *sDscamα* and *Dscamβ* genes from various species. Interestingly, the number of 5′ cassette tandem duplicates per gene correlated inversely with the size of the duplicate blocks in *M. occidentalis*, *I*. *scapularis*, *M*. *martensii*, and *S. mimosarum* (Fig. 4e−h). These correlations largely fitted to a power law. *L. polyphemus* did not exhibit as strong a correlation as the other species (Fig. 4i), possibly due to the incomplete annotation of the *Dscam* genes. Furthermore, the numbers of 5′ cassette duplicates per *sDscamα* were roughly 1–3-fold higher than those in *sDscamβ*s, with the duplicate blocks containing two additional exons than those in *sDscamα*s (Fig. 4j)*.* This inverse relationship might reflect an inherent property of the species, i.e., the genome size (Fig. 4k, l). For example, *I*. *scapularis*, with a large genome size estimated at 2100 Mb [29], contained more *sDscam* gene duplicates, but fewer 5′ cassette tandem duplicates in each gene. In contrast, *M. occidentalis*, with a small genome size (~152 Mb), had fewer *sDscam* gene duplicates, but more 5′ cassette tandem duplicates per gene. In particular, phylogenetic analysis indicated loss of cassette-within introns occurred independently in *sDscamβ1* and *sDscamβ2* of *M. occidentalis*, whereas no substantial intron loss occurred in the constant region (Fig. 4m). This result led us to speculate that intron loss caused the decreased repeat block size to facilitate greater duplication in the 5′ variable region. Conversely, this species-specific intron loss might be driven by the selection pressure of greater cassette duplication. These results suggest that gene duplications and 5′ cassette tandem duplication are not selected to expand independently of each other during Chelicerata evolution.

### Differential and biased expression of nonclassical Dscam isoforms

**Fig. 5:**
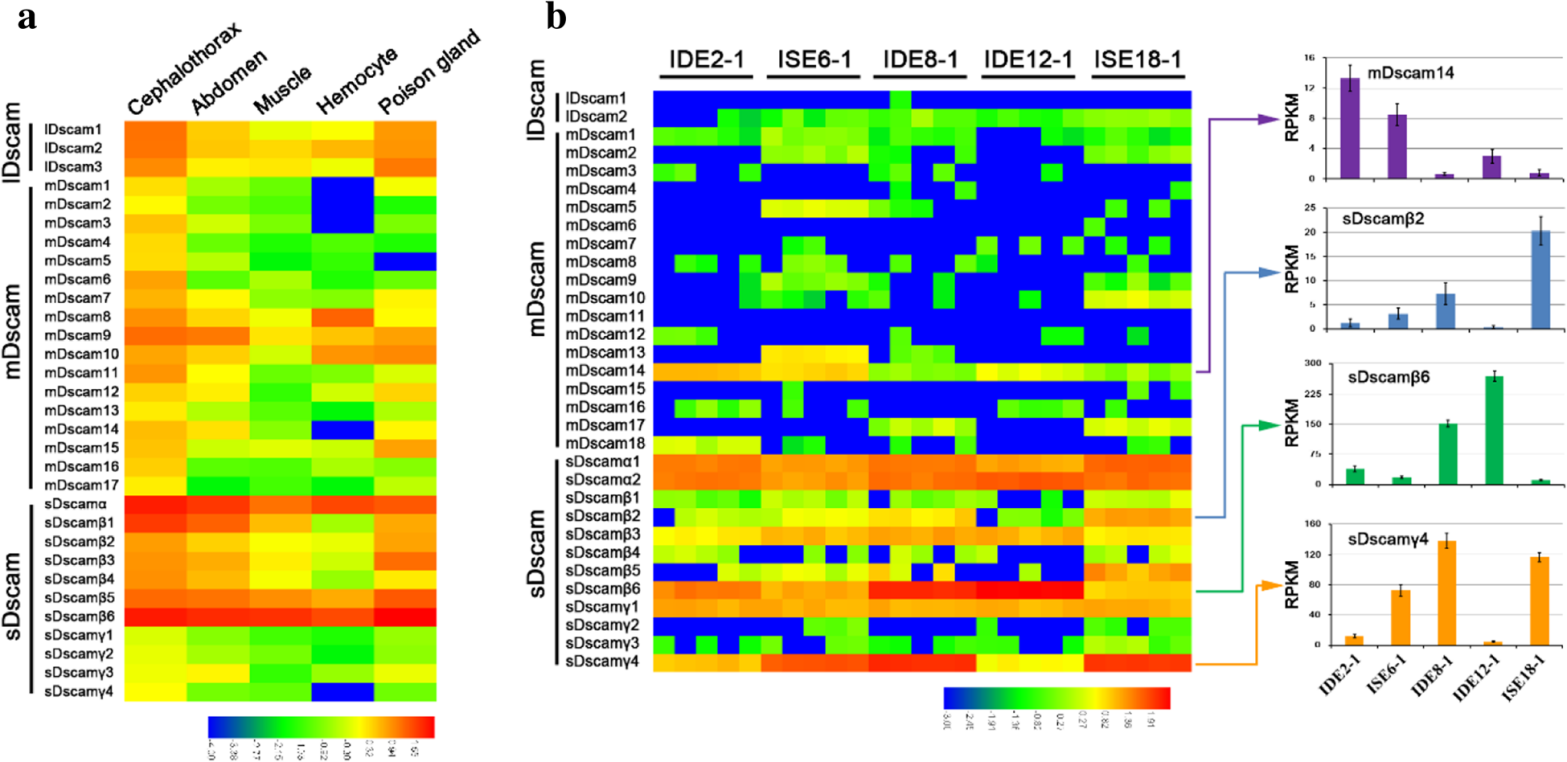
Differential expression of nonclassical *Dscam* genes. **a** Heatmap of expression of 31 *Dscam* genes in various tissues of *M*. *martensii*. The expression level for each transcript is shown as reads per kilobase of transcript per million mapped reads (RPKM) of its corresponding constitutive exons. The 25-nt fragmented RNA-sequencing datasets were mapped to calculate the relative expression level. The maximum expression levels of *mDscam8* were found in hemocytes, whereas *mDscam10* and *mDscam15* were highly expressed in poison glands. **b** The differing expression patterns of *Dscam*s in *I*. *scapularis* lineages. This result indicates that many *Dscam*s were preferentially and specifically expressed in certain lines. Data are expressed as the mean ± SD from three independent experiments

To further characterise the expression of nonclassical Dscam isoforms, we employed RNA-seq data to analyse the expression of the nonclassical *Dscam*s in various tissues of *M*. *martensii*. Similar to *sDscamα* and *sDscamβ* [25], as well as three classical *Dscam*s (*LDscams1*–*3*), 14 of 17 *mDscam*s and 3 of 4 *sDscamγ*s were expressed at markedly higher levels in the cephalothorax than in other tissues (Fig. 5a; Additional file 3: Figure S5). This expression pattern is largely coincident with high expression of classical Dscams in the nervous system of vertebrates and insects [5, 30–32]. In contrast, *LDscam*s, *mDscam*s, and *sDscam*s showed low expression in muscles. Notably, *mDscam8* was specifically expressed at maximum levels in haemocytes, whereas *mDscam10* and *mDscam15* were highly expressed in poison glands (Fig. 5a). It will be interesting to see whether nonclassical *Dscam*s play a potential role in Scorpione immunity, similar to that of *Dscam1* isoforms in the *Drosophila* immune system [5]. These results suggest that nonclassical *Dscam* expression is regulated spatially and temporally.

The wealth of published RNA-seq data from a set of 20 embryo-derived cell lines allows the comparison of *Dscam* expression in various cell lines of *I*. *scapularis* (Additional file 4: Table S3). Expression profiling revealed that *Dscam* genes were differentially expressed in various cell lines (Fig. 5b). Interestingly, many *Dscam*s were preferentially and specifically expressed in certain lines. For example, high expression of *mDscam14* was observed in strain IDE2, but was almost undetectable in strain IDE8 and ISE18. In contrast, *sDscamγ4* showed robust expression in strain IDE8 and ISE18, but low level expression in strain IDE12; *sDscamβ2* showed high expression in strain ISE18. Likewise, the 5′ variable exons of *sDscamα* and *sDscamβ* genes exhibited differential expression in various lineages (Additional file 3: Figure S6). *sDscamα2.14* was specifically expressed at maximum levels in strain ISE6, while *sDscamβ2.12* and *sDscamβ6.6* were expressed at maximum levels in strain ISE18 and IDE12, respectively. Collectively, these results revealed lineage-specific expression signatures of Dscam isoforms.

**Fig. 6:**
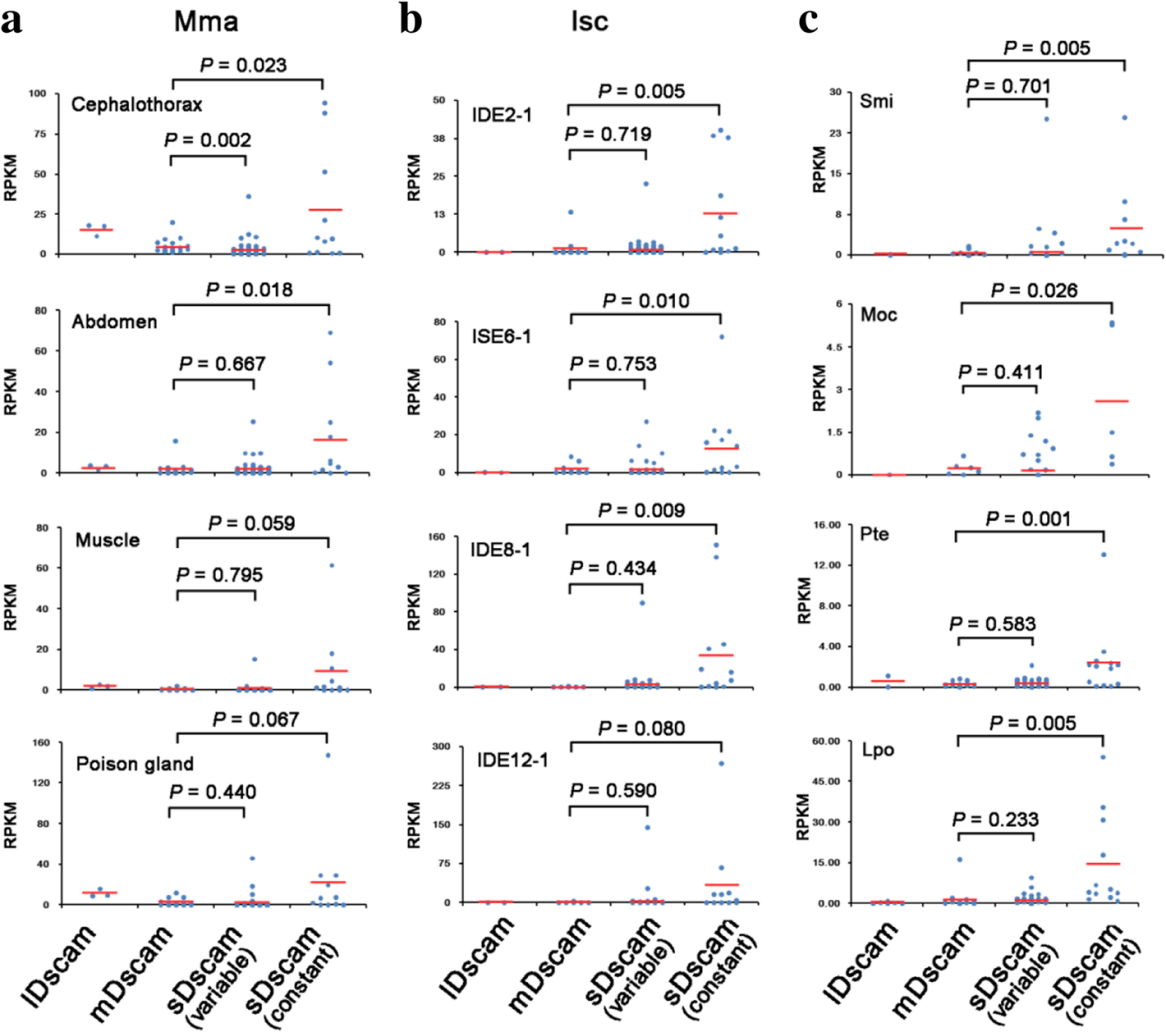
*sDscam* genes are biased to be more highly expressed. **a** In *M*. *martensii*, *sDscam* genes are biased to be more highly expressed than *mDscam* genes*.* We calculated p values using a two-tailed Student’s *t*-test. As *LDscams* occurs much less frequently than *mDscams* and *sDscams*, we did not analyze the statistical differences between them. **b** *sDscam* genes are biased to be more highly expressed than *mDscam* genes in *I*. *scapularis*. **c** The expression bias is conserved in *S. mimosarum* (Smi, SRR1015314), *P. tepidariorum* (Pte, SRR1824487), *M. occidentalis* (Moc, SRR446504), and *L. polyphemus* (Lpo, SRX1323743)

We observed a dramatic bias: *sDscam*s were largely expressed at higher levels than *LDscam*s and *mDscam*s in various tissues of *M*. *martensii* (Fig. 6a), and in different *I*. *scapularis* lineages (Fig. 6b). A similar bias was observed in *S. mimosarum*, *P. tepidariorum*, *M. occidentalis*, and *L. polyphemus* (Fig. 6c). These data indicate that *sDscam*-biased expression patterns are evolutionally conserved across Chelicerata. It is possible that the high expression of *sDscam*s might be due to the use of multiple promoters, leading to more transcripts. Taken together, both the number and expression level of nonclassical *Dscam*s dominate exclusively in the Dscam isoform repertoire across Chelicerata.

## Discussion

### The evolutionary landscape of Dscam diversity in arthropods

This study identified large and diverse nonclassical Dscam repertoires in chelicerates, most of which are newly annotated or corrected. The results reported here extend our previous insights into Dscam diversity during arthropod evolution [23–25]. Firstly, the nonclassical *Dscam* genes can be classified into four types (*mDscam*, *sDscamα*, *sDscamβ*, and *sDscamγ*) based on their size and structure (Fig. 1). Moreover, the nonclassical *Dscam* genes are conserved and restricted to Chelicerata, suggesting that Chelicerata uniquely evolved a large lineage-specific repertoire of nonclassical Dscam isoforms. Phylogenetic analysis and comparison of Dscam structures revealed that the *Dscam* ancestor underwent multiple shortening events during chelicerate evolution, leading to the loss of protein domains to varying degrees [25]. Thus, the *Dscam* gene number has undergone massive expansion, and also the structure of *Dscam* genes became highly diversified during Chelicerata evolution. These findings, together with those of others [23–25], provide a global framework for the evolution of Dscam diversity in arthropods.

These Dscam diversifications suggest that their binding mechanisms are potentially different from those of classical Dscams. The crystal structural analysis reveals that the first four Ig domains of *Drosophila* Dscam1 are responsible for the formation of the horseshoe configuration [33]. Additional modelling studies illuminated the molecular basis of the isoform-specific homophilic binding specificity [9]. As they contain the same eight N-terminal Ig domains as *Drosophila* Dscam1, it is conceivable that the chelicerate mDscams studied in this research could form a horseshoe configuration and interact via a similar mechanism. However, this horseshoe structure does not form in chelicerate sDscams with only three Ig domains. Therefore, we speculate that chelicerate sDscams exhibit a different mode of isoform-specific homophilic binding than *Drosophila* Dscam1.

Chelicerata seem to have generated far fewer Dscam isoforms than insects. As estimated by the number of Ig7 or orthologues, the number of Dscam isoforms is in the range of ~100–200 across the Chelicerata species investigated (Fig. 1), approximately two to three orders of magnitude lower than that in insects. Recent studies indicate that clustered mammalian Pcdhs could expand the binding specificity repertoire via *cis*-multimers. For 22 γ-Pcdhs, the diversity of adhesive interfaces could be on the order of 10^5^ through *cis*-tetramerisation coupled with homophilic *trans* interactions [34, 35]. Given the striking organisational resemblance between the Chelicerata clustered *sDscam*s and mammalian *Pcdh*s, it is attractive to speculate that Chelicerata sDscams could function via *cis*-multimers. If so, the diversity of adhesive interfaces mediated by Chelicerata sDscams will be much higher, as there are many more Chelicerata-clustered *sDscam*s (90–130) than mammalian-clustered *Pcdh*s (50–60).

### Dscams versus Protocadherins

Dscams and Pcdhs belong to large established families of cell adhesion molecules: Dscams belong to the Ig superfamily and Pcdhs belong to the cadherin superfamily [36]. The clustered Pcdh diversity is confined within the clade of jawed vertebrates, which is considered as a chordate innovation [37], whereas extensive Dscam diversity is unique to arthropods. In the latter case, arthropods diversify using two mechanisms to generate Dscam isoform diversity: Mandibulata *Dscam* genes employ exclusive splicing of internal exon clusters to generate distinct isoforms [3, 24] and chelicerate *Dscam*s utilise alternative promoters in the 5′ variable region [25]. However, neither cadherin nor protocadherin genes appear to have internal tandem exon arrays in a manner similar to Mandibulata *Dscam*s.

**Fig. 7:**
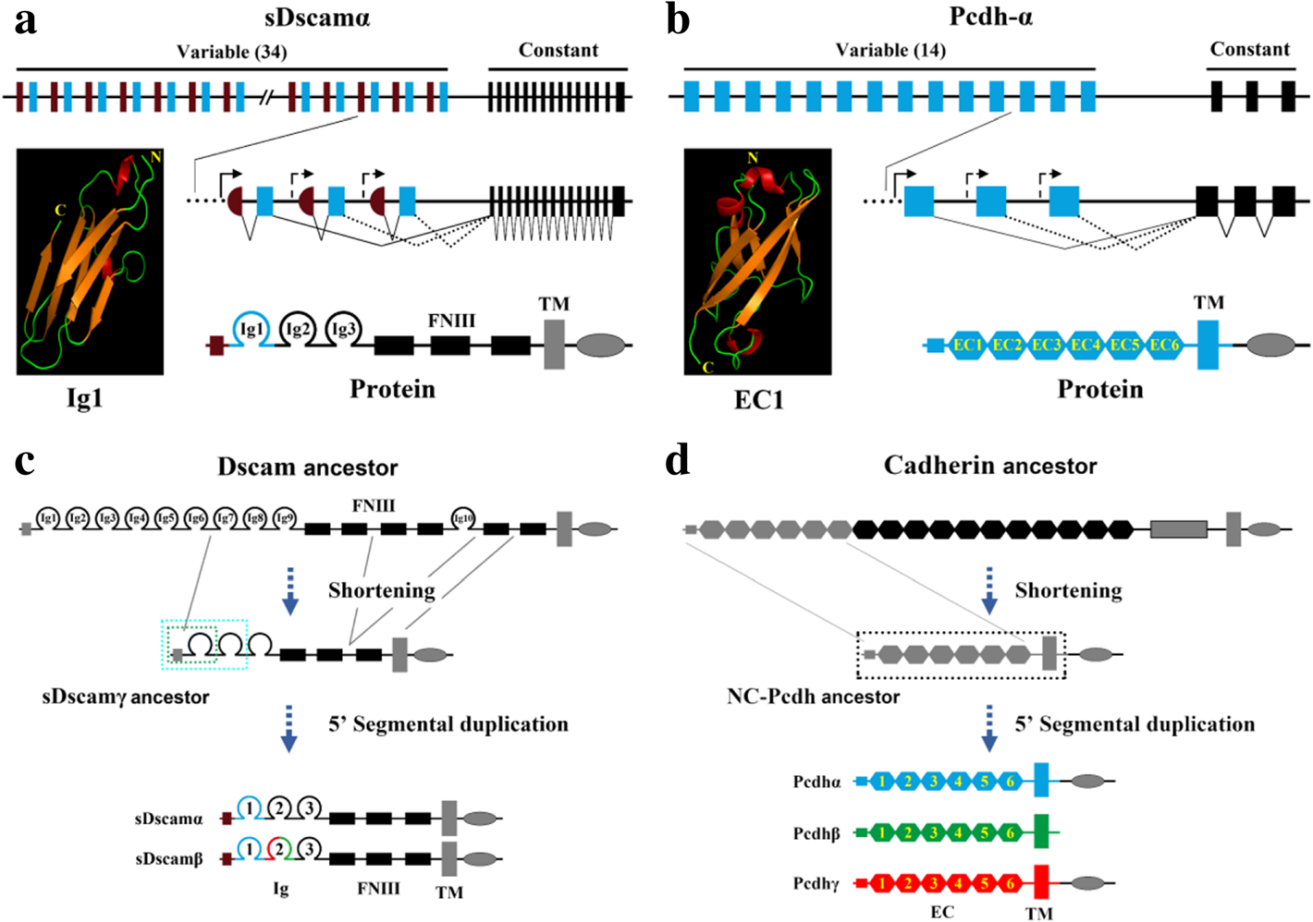
Comparisons of organisation and origin of clustered *Dscam* and *Pcdh* genes. **a** Schematic diagram for the *sDscamα* gene in Chelicerata. Symbols used are the same as in Fig. 1. Each variable cassette was transcribed by an alternative promoter followed by alternative splicing. The variable cassette encoded the N-terminal Ig1 domain (blue). Tertiary structure model of Ig1 of *I*. *scapularis* sDscam is shown on the left. **b** Schematic diagram for the *Pcdhα* genes in vertebrates. The Pcdh gene cluster contains exons that encode 14 extracellular and TM domains [18]. Each repeat is preceded by a promoter, and encodes extracellular and TM domains. Tertiary structure model of the EC1 domain of Pcdhα is shown on the left, which is similar to that of the Ig1 domain of sDscam. **c, d** Comparisons between the evolutionary origin and expansion of clustered *Dscam* (c) and *Pcdh* (d) genes. The 5′ clustered organisation of both *sDscam* and *Pcdh* genes may originate from the shortening and expansion of the ectodomains of canonical *Dscam* via sequential duplication and mutation

Both clustered chelicerate *Dscam*s and vertebrate *Pcdh*s are organised in a tandem array in the 5′ variable region (Fig. 7a, b). Curiously, both 5′ clustered *sDscam*s and *Pcdh*s appeared to originate via an analogous evolutionary pathway (Fig. 7c, d), which was involved in the shortening and expansion of *Dscam* and *cadherin* ancestors [25, 38]. In both genes, each variable repeat is preceded by a promoter, and differential expression occurs via combining alternate promoter choice with alternative splicing [19, 20, 25]. Moreover, 5′ clustered *Dscam*s and *Pcdh*s contain similar structural composition encoding six extracellular domains, a single transmembrane (TM) region, and a cytoplasmic domain. Interestingly, three-dimensional protein structure modelling revealed a similar β sandwich structure between the first domain of Pcdh and sDscam (Fig. 7a, b). Finally, we showed that clustered *sDscam*s encode proteins exhibiting isoform-specific homophilic binding in a manner similar to *Pcdh*s. Despite their overall similarities, the structural properties of *Pcdh* and *sDscam* genes differ in at least two major aspects. First, inconsistent with the conserved arrangement of a single genome locus containing three tandem *Pcdh* gene clusters across vertebrates, the *sDscam*s are largely dispersed or partially clustered in the chelicerate genome. As the transcription of *Pcdh* gene clusters is closely linked and mediated by long-range chromatin-looping interactions [39], the dispersed distribution of *sDscam* gene clusters might reflect a mode of transcription regulation distinct from that of *Pcdh* genes. A second major difference concerns the structure of the variable region. *Pcdh* variable exons encode the entire ectodomain composed of six extracellular cadherin domains (EC1–EC6), a single TM region, and a short cytoplasmic extension, whereas the variable cassettes of *sDscam*s encode the partial ectodomain of one or two Ig domains at the N-terminus. Future studies are needed to investigate the role of variable region structures in the subcellular distribution, isoform-specific binding, and multimer formation.

Combining experimental evidence with the complementary phylogenetic distribution of Dscam diversity in arthropods and Pcdh diversity in vertebrates, it is tempting to suggest that both may have similar roles in the nervous system [15–17, 21, 22]. These two phyla seem to employ a similar strategy for self/non-self discrimination by recruiting various molecules of different protein families [15]. Nevertheless, there is a wide evolutionary gap between arthropods and vertebrates, as they share a common ancestor more than 500 million years ago. It will be informative to explore the molecules or mechanisms of species within this phylogenetic gap (i.e., *Branchiostoma floridae*), which are thought to lack both clustered *Pcdh* and *Dscam* genes, and that have evolved to endow cells with distinct molecular identities and highly diverse recognition selectivity strategies.

### Diversification of Ig superfamily protein repertoires

Although tandemly arrayed Ig repeats are found frequently in the genome, animals achieve protein diversity from a single locus via a variety of mechanisms (Additional file 3: Figure S7). In higher vertebrates, the great diversity of antigen-specific receptors of the adaptive immune system can be achieved through somatic gene rearrangement and clonal selection at the DNA transcriptional level, which is known as the V(D)J mechanism [40–43]. However, insect *Dscam1*s utilise mutually exclusive splicing to generate an extensive repertoire of thousands of Ig-superfamily protein isoforms [3, 5]. In contrast, our studies indicate that Chelicerata *sDscam*s employ alternative promoters to generate substantial numbers of isoforms [25]. These molecular processes are mutually exclusive among three distinct clades, functioning to create extensive molecule diversity. Metazoans might have evolved different ways for extensive Ig-superfamily proteins to enable immune defence and other functions.

## Conclusions

In this study, we identified large and lineage-specific nonclassical Dscam repertoires in chelicerates. Nonclassical *Dscam*s are conserved and restricted to Chelicerata, and have been classified into four types based on their size and structure. These results demonstrate that arthropods specifically diversify a large Chelicerata-specific repertoire of nonclassical Dscam isoforms. The Dscam isoform repertoire across Chelicerata is dominated exclusively by the number and expression levels of nonclassical *Dscam*s. This genome-wide identification and classification study of *Dscam* genes provides the global framework of the evolution of Dscam diversity in arthropods, and provides mechanistic insights into the diversification of the species-specific Ig superfamily repertoire.

## Methods

### Data availability and RNA-seq data analysis

We investigated the following representative Chelicerate species: Mesostigmatan *M. occidentalis*, Trombidiformes *I*. *scapularis* [29], two Araneae *S. mimosarum* and *P. tepidariorum* [44], two Scorpiones *M*. *martensii* [45], and Merostomatan *L. polyphemus.* The sources of the Chelicerata genome sequences used in this study are shown in Additional file 1: Table S1. To validate the *Dscam* candidates, we selected 125 publically available RNA-seq datasets corresponding to various developmental stages, tissues, organs, and cell lines across six chelicerate species (Additional file 4: Table S3). All of the raw RNA-seq datasets were subject to pre-treatment, including adapter trimming and low-quality read removal using the FASTX-Toolkit (http://hannonlab.cshl.edu/fastx_toolkit/index.html). Then, for each sequencing dataset, the count of each RNA-seq read was normalized to reads per million (RPM), thus enabling cross-sample comparison of the *Dscam* expression levels. Specifically, to calculate the RPM of one RNA-seq read, the raw count of the read was divided by the total raw count of the RNA-seq dataset and multiplied by 10^6^. The treated reads were mapped onto the transcripts using Bowtie 2 [46]. From these mappings, we were able to calculate the expression levels in reads per kilobase of transcript per million mapped reads (RPKM).

### Availability of genome and RNA-seq data

We investigated the following representative species of Chelicerate: Mesostigmatan *M. occidentalis*, Trombidiformes *I*. *scapularis* [29], two Araneae *S. mimosarum* and *P. tepidariorum* [44], two Scorpiones *M*. *martensii* [45], and Merostomatan *L. polyphemus.* The sources of the Chelicerata genome sequences used in this study are shown in Additional file 1: Table S1. For *Dscam* candidate validation, we selected 125 publically available RNA-seq data corresponding to various developmental stages, tissues, and organs, and cell lines across six chelicerate species (Additional file 4: Table S3).

### Annotation and identification of *Dscam* genes

The sequences of the *Dscam* homologues were annotated through cross-species BLAST searches using the available annotated *Dscam* sequences (http://blast.ncbi.nlm.nih.gov/Blast.cgi). These *Dscam* candidate homologues were validated further using publically available transcriptome and RNA-seq datasets. All *Dscam* candidates were confirmed by phylogenetic analysis (http://www.ebi.ac.uk/clustalw/index.html) and then analysed by classifying and predicting protein domains with InterPro [47] (http://www.ebi.ac.uk/interpro/) and PROSITE [48] (http://prosite.expasy.org/prosite.html). The *Dscam* genes identified in representative species of Chelicerate are listed in Additional file 2: Table S2.

### Phylogenetic analysis

The *Dscam* sequences were aligned across species using the Clustal W2 software package (http://www.ebi.ac.uk/clustalw/index.html) [49]. The coding sequences of the variable region were translated, and the resulting polypeptides were aligned. The nucleotide sequences of each 5′ variable cassette of *sDscam* were translated into amino acid sequences and aligned. The genetic distances for each gene were estimated using MEGA 7.0 software [50]. We used maximum likelihood (ML) methods, using MEGA [50], to build the phylogenies. For the ML analysis, we ran MEGA with at least 1000 bootstrap replicates. To determine the homology of the Dscam-related genes found in metazoans, we estimated the phylogenies of 217 proteins, including Dscams encompassing the seventh Ig domains to the end (sDscam regions encompassing the first Ig domains to end) (Fig. 2). This phylogeny was rooted using the sequence of the *Nematostella vectensis* Dscam (GenBank:ABAV01020293.1) [51]. We used the duplicated cassettes encoding the Ig1 domain of *sDscamα* to dissect the evolutionary relationships between the duplicated cassettes with a tree rooted on the *D. melanogaster Dscam1* duplicated exon 9.1, which encodes the Ig7 domain (Additional file 3: Figure S3a). Similarly*,* we used the duplicated cassettes, which encode the Ig1 + 2 domain of *sDscamβ* and a tree rooted on the *D. melanogaster Dscam1* exon 9.1, 10, and 11, which encode the Ig7 + 8 domains (Additional file 3: Figure S3b).

### Protein three-dimensional structure modelling

All three-dimensional proteins structures were acquired using the Swiss-Model (automated mode) (www.swissmodel.expasy.org). The structures were displayed and processed using the PyMOL software package (www.pymol.com).

### Analysis of differential and biased expression

The RNA-seq data from publically available samples were used to analyse the expression of nonclassical *Dscam* genes at various developmental stages, tissues types, and cell lines (Additional file 4: Table S3). For each sample, we calculated the RPKM value of the constant exonic region to measure the expression level of each *Dscam* gene from the replicates. The alternative exon encoding Ig7 was selected to calculate the expression level from the replicates for each 5′ variable cassette. Considering the short length of the alternative exons, the RNA-seq reads were split into 25-nucleotide (nt) fragments for mapping by using Bowtie 2 software [46], and only the perfectly mapped fragments were retained for expression level calculation. Furthermore, the read counts of a 25-nt fragment with multiple loci were divided by the number of loci, and then assigned equally to each locus for expression level calculation. To eliminate influences on calculations of the expression levels from identical sequences among exon duplicates, both the 25-nt fragments and the full-length RNA-seq reads (150 nt) were used to calculate the expression profiles as previously described [25]. If, as a result of originating from a repetitive region, one RNA-seq read or fragment mapped onto several loci, we divided the RPM value of this RNA-seq read by the number of repetitive loci, then evenly assigned it to each transcript in the expression level calculation.

### Statistical analysis

We used an independent sample *t*-test to assess the relationship between the 5′ cassette duplicate and the sDscam gene duplicate. We compared the numbers of 5′ cassettes and gene duplicates between the sDscamα and sDscamβ groups using a two-tailed Student’s *t*-test. We defined correlation and significance levels for the number of 5′ cassette duplicates and the repeat size in terms of a simple regression model. We also used a two-tailed Student’s *t*-test to compare the differences between the expression levels in groups of mDscams and sDscams across various species. Effects were considered to be statistically significant when *p* < 0.05.

## Additional files

Additional file 1: Table S1. Chelicerata species and their genome sources in this study. (XLSX 10 kb)

Additional file 2: Table S2. A list of *Dscam* homologues identified in Chelicerata species. (XLSX 25 kb)

Additional file 3: Supplementary Figures S1–S7. (PDF 1532 kb)

Additional file 4: Table S3. RNA-seq datasets in Chelicerata species. (XLSX 21 kb)

## Abbreviations

BLAST: Basic local alignment search tool
Dscam: Down syndrome cell adhesion molecule
EC: Extracellular cadherin domains
FNIII: Fibronectin III
Ig: Immunoglobulin
Isc: *Ixodes scapularis*
LDscam: Large Dscam
Lpo: *Limulus polyphemus*
mDscam: Middle Dscam
MEGA: Molecular evolutionary genetics analysis
Mma: *Mesobuthus martensii*
Moc: *Metaseiulus occidentalis*
mRNA: Messenger RNA
Pcdhs: Protocadherins
Pte: *Parasteatoda tepidariorum*
RNA-seq: RNA Sequencing
RPKM: Reads per kilobase of transcript per million mapped reads
RPM: Reads per million
sDscam: Shortened Dscam
Smi: *Stegodyphus mimosarum*
TM: Transmembrane
